# Autophagy Modulators From Chinese Herbal Medicines: Mechanisms and Therapeutic Potentials for Asthma

**DOI:** 10.3389/fphar.2021.710679

**Published:** 2021-07-23

**Authors:** Yun Zhang, Xing Wang, He Zhang, Hongmei Tang, Hang Hu, Songping Wang, Vincent Kam Wai Wong, Yuying Li, Jun Deng

**Affiliations:** ^1^ Inflammation and Allergic Diseases Research Unit, The Affiliated Hospital of Southwest Medical University, Luzhou, China; ^2^ Department of Respiratory and Critical Care Medicine, The Affiliated Hospital of Southwest Medical University, Luzhou, China; ^3^ Dr. Neher′s Biophysics Laboratory for Innovative Drug Discovery, State Key Laboratory of Quality Research in Chinese Medicine, Macau University of Science and Technology, Macau, China

**Keywords:** Chinese herbal medicines, autophagy, asthma, inflammation, mucus hypersecretion

## Abstract

Asthma has become a global health issue, suffering more than 300 million people in the world, which is a heterogeneous disease, usually characterized by chronic airway inflammation and airway hyperreactivity. Combination of inhaled corticosteroids (ICS) and long acting β-agonists (LABA) can relieve asthma symptoms and reduce the frequency of exacerbations, especially for patients with refractory asthma, but there are limited treatment options for people who do not gain control on combination ICS/LABA. The increase in ICS dose generally provides little additional benefit, and there is an increased risk of side effects. Therefore, therapeutic interventions integrating the use of different agents that focus on different targets are needed to overcome this set of diseases. Some findings suggest autophagy is closely correlated with the severity of asthma through eosinophilic inflammation, and its modulation may provide novel therapeutic approaches for severe allergic asthma. The chinese herbal medicine (CHM) have been demonstrated clinically as potent therapeutic interventions for asthma. Moreover some reports have found that the bioactive components isolated from CHM could modulate autophagy, and exhibit potent Anti-inflammatory activity. These findings have implied the potential for CHMs in asthma or allergic inflammation therapy via the modulation of autophagy. In this review, we discuss the basic pathomechanisms underpinning asthma, and the potential role of CHMs in treating asthma with modulating autophagy.

## Introduction

Asthma has become a global health issue affecting approximately 4% of the world population. The number of patients suffering from the disorder is expected to reach 400 million worldwide by 2025 (Nunes et al., 2017). According to a cross-sectional questionnaire survey of children asthma and other allergies (1–8 years old) conducted in ten cities of Mainland China, juvenile asthma cases were increasing from 1990 to 2011, which demonstrated an aggravating trend since 2000 (Zhang et al., 2013). Conventional therapies for asthma control include the application of steroids, for example, inhaled corticosteroids (ICS) or the combination of ICS and long-acting β2 agonist (LABA). However, insensitivities towards such treatment strategies are frequently observed, partially due to the development of steroid resistance, which progressively evolves into severe asthma (Sin et al., 2004; Ni Chroinin et al., 2005). As a result, the quality of life of patients is worsened with increased expense of hospitalizations, as well as higher risk of asthma attacks and death (Fergeson et al., 2017). Approximately 5–10% of asthma patients are affected by severe and persistent asthma (Lang, 2015). In addition, long-term exposure to ICS may affect the height and weight of children. Therefore, novel and reliable pharmaceutical interventions are needed for the effective control of asthma, particularly for those with established steroid resistance (Christensson et al., 2008).

Autophagy, a cellular self-engulfing process employed for the removal of unwanted organelles and misfolded proteins, plays a critical role in the pathogenesis of a variety of inflammatory disorders, including respiratory illness diseases like asthma (Jyothula and Eissa, 2013). Intriguingly, some reports have found that the bioactive components isolated from Chinese herbal medicine (CHM), such as celastrol, a constituent of the CHM could modulate autophagy, and exhibit anti-inflammatory effects (Hu et al., 2017; Liu et al., 2019). Such findings suggest a potential for CHMs in asthma or allergic inflammation therapy via the modulation of autophagy. In fact, some clinical studies have revealed that CHM decoctions can control asthma progression with significant efficacy, and CHMs are the ideal source for autophagy modulators (Law et al., 2016). In this review, we will briefly discuss the basic pathomechanisms underpinning asthma, specifically focusing on the role of autophagy and the autophagic process. The traditional medication for treating the disease and the clinical limitations that plague their therapeutic efficacy will also be described. And, we also discuss CHMs with potential for asthma therapy, autophagy regulatory mechanisms and their role in human diseases. Further discussion on specific CHMs will focus on their new therapeutic usage through regulation of autophagy.

## Mechanisms and Regulation of Asthma Progression Involved in Autophagy

Asthma is a heterogeneous disease with several underlying disease processes, usually characterized by chronic airway inflammation, airway hyperreactivity and airway remodeling. The classic allergic airway inflammation is caused by submucosal infiltration of activated T-lymphocytes, eosinophils, neutrophils, epithelial cells, macrophages and mast cells (Adcock and Caramori, 2001). Thus, initialing the allergic airway inflammation process is a complex web of cells and cell signaling molecules interacting to elicit an inflammatory response. Some pathological and physiological evidence reported that the airway inflammatory process could extend from the central airway to the peripheral airways and the lung parenchyma (Trevor and Deshane, 2014). Also asthma is typically associated with an imbalance between Th1 and Th2 pathways; over-driven Th2-mediated inflammation leads to airway inflammation and airway remodeling (KleinJan, 2016). More over eosinophils play important roles in augmenting AHR, mucus production, and airway remodeling in allergic asthma by producing IL-13 and leukotrienes from eosinophil lipid bodies (George and Brightling, 2016). Also eosinophils contribute to much of the damage of the respiratory epithelium during the late phase of asthma. The activation and granulation of eosinophils is also related to the activation of the alveolar macrophages (McBrien and Menzies-Gow, 2017). Eosinophils also release cesteinyl leukotrienes and reactive oxygen species resulting in airway inflammation and mucus secretion in the asthma response. Present studies have shown that autophagy participates in the immunopathology of inflammatory diseases and plays role in eosinophil inflammation in asthma (Silveira et al., 2020). Thus, asthma inflammation progression most partly involved in autophagy. And a role for autophagy in eosinophils inflammation in asthma usually targeted in severe asthma (Liu et al., 2016). Therapeutic strategies to target autophagy may provide a new approach to treating asthma airway inflammation.

The extensive infiltration of eosinophils into the airway is a hallmark of allergic asthma. The numbers of eosinophils in peripheral blood, bronchoalveolar lavage fluid (BALF), and epithelium were not only associated with lung function but also associated with the severity of asthma (Bousquet et al., 1990). Eosinophils with inflammatory lesions in the lungs produce and release a variety of proinflammatory mediators, including basic proteins (major basic protein, eosinophil cationic protein [ECP], eosinophil peroxidase, eosinophil-derived neurotoxin), cytokines (IL-2, IL-3, IL-4, IL-5, IL-10, IL-12, IL-13, IL-16, and IL-25), chemokines (CCL5, CCL11, and CCL13), growth factors (TNF and TGF-α/β) (Hogan et al., 2008; Liu et al., 2016). IL-13 might be implicated as a mediator of tight junction disruption in asthma (Georas and Rezaee, 2014). Bronchial epithelium barrier dysfunction is the central process involved in the development of the onset of asthma pathophysiology (Holgate, 2008; Fujita et al., 2011). The fragile epithelium barrier in asthmatic patients manifests more airway inflammation and bronchial hyper-responsiveness. Conversely, epithelial barrier dysfunction also contributes to Th2-type cell-mediated immunity. Among these cytokines TGF-β focused on progression of airway remodeling including mucus hyper-secretion in asthma associated with autophagy, inhibition of autophagy exerted therapeutic benefits for TGF-β3 induced airway mucus secretion (Zhang et al., 2018).

Many researchers have reported that there is a subtype of severe asthma with persistent symptoms and high risk of exacerbation. IL-17 cytokines released by Th17 cells mediate severe asthma inflammation via augmentation of expression of GR-β, which inhibits the action of glucocorticoid receptor GR-α Silverpil and Lindén (2012) leading to steriod resistant. The overwhelming lung production of ROS initiates severe airway inflammation associated with its direct damage to DNA in bronchial epithelial cells (Chan et al., 2016; Zhang et al., 2020). ROS can not only induce oxidative DNA damage but also induce DNA double strand breaks in epithelial cells in asthma. A previous study has demonstrated that DNA repair inhibition significantly augments IL-4, IL-5, IL-13, and IL-33 levels in epithelial cells exposed to house dust mites (HDM) (Chan et al., 2016). Chronic inflammation leads to a key feature of severe asthma, which is characterized by airway remodeling. Airway remodeling is the modification of the normal structural properties of the airway wall that plays an important role in pulmonary dysfunction in asthma (Naylor, 1962; Laitinen et al., 1985). Mucus secretion is a tipical characteristic of airway remodeling involved in PI3K/Akt pathway activity and subsequently enhance endoplasmic reticulum stress (ER stress) (Wang et al., 2017). Mucociliary clearance is critically important in protecting the airways. But overwhelming of mucus may be result in acute and chronic effects dysfunction of mucociliary clearance, which is known to caused Oxidative stress (O'Grady, 2019). Current asthma therapies fail to target airway remodeling that correlates with asthma severity driving disease progression that ultimately leads to loss of lung function (McAlinden et al., 2019). The influence of oxidative stress and autophagy on airway showing the potential of autophagy modulators in restoring the function of effective mucociliary clearance (O'Grady, 2019). Lack of autophagy causes a severe IL17-mediated neutrophilic lung inflammation of asthma (Suzuki et al., 2016). These demastrated that autophagy pathway in the lung might be an effective therapeutic target for patients with refractory severe asthma. How autophagy affects asthma inflammation and airway remodeling is worthing to excavating.

## Role of Manipulating Autophagy in the Pathogenesis of Asthma

Autophagy is a highly conserved homeostatic mechanism for cell survival under conditions of stress, and is widely implicated as an important pathway in many biological processes and diseases (Levine and Kroemer, 2008; Choi et al., 2013). The normal function of autophagy is protein degradation and turnover of destroyed cell organelles for new cell formation. In the most common form of autophagy, cytosolic materials are sequestered into double-membrane compartments called autophagosomes, which subsequently fuse to lysosomes wherein their contents are enzymatically degraded [36]. Recently, autophagy has gained more attention in human pulmonary diseases, both as a modulator of pathogenesis and as a potential therapeutic target (Chen et al., 2016; Dickinson et al., 2016; Zhou et al., 2016). A number of autophagy-related proteins (ATGs), together with other proteins, are involved in the process of forming autophagosomes, starting from the formation of the autophagy initiation complex to the elongation of autophagosome membranes (He and Klionsky, 2009). The ATG proteins can be classified into several functional units: the ULZK complex, ATG9L, the class III PI(3)K complex, the ATG2-WIPI complex, the ATG12 conjugation system, and the LC3 conjugation system. The ULZK complex is the most upstream component in the autophagy pathway, which is required for recruitment of the autophagy-specific class III PI(3)K complex. LC3 is widely used as a marker for the microscopic detection of isolation membranes and autophagosomes. The amount of LC3-II is also widely used for the quantification of autophagic activity. Autophagy has been studied in relation to infection by bacteria, viruses, and parasites (Choi et al., 2013). At present, accumulating evidence has revealed the relationship between autophagy and inflammation, especially the effect of autophagy on chronic inflammation in lung disease (Wesselborg and Stork, 2015).

### Autophagy in Inflammation

Allergic asthma is mediated by TH2 cells and type two innate lymphoid cells (ILC2) for production of inflammatory cytokines IL-5 and IL-13 (Bartemes et al., 2014; Lewis et al., 2019). Airway eosinophilic inflammation can cause mild-to-moderate asthma; however, neutrophils are often predominant in the sputum of patients with severe asthma having corticosteroid resistance (Pham et al., 2017). Autophagy plays a role of neutrophil survival and the formation of neutrophil extracellular DNA traps which resulting from neutrophils activating (Remijsen et al., 2011; Brinkmann and Zychlinsky, 2012). Elevated neutrophil autophagy and NET productions in patients with asthma could enhance airway inflammation. On the contrary, in the peripheral blood neutrophils in asthma could be primed by pro-inflammatory and inflammatory cytokines to induce a high level of autophagy that could maintain or enhance neutrophil activation. Autophagy also has an effect on eosinophil activation and effffector function. Conversely, eosinophils activation by the presence of IL-5 induces autophagy further leading to inflammation (Ban et al., 2016).

However, in pulmonary dendritic cells, asthma could reduce the numbers of LC3 foci. Atg5 deficency mice were found to have no decrease in AHR showing a steroid resistant phenotype with increase concentrations of IL-1 and IL-23 further contributing to TH17 neutrophilic polarity (Suzuki et al., 2016). In macrophages, the alleviation of autophagy was associated with increased inflammation (Liu et al., 2015a). Consistently, previous studies reported that hampered autophagy, through blocking of Atg5/7, LC3, and Beclin-1, led to increases in IL-1β and IL-18, suggesting a possible protective role of autophagy in inflammatory contexts (Zhang et al., 2012; Pu et al., 2017; Choi et al., 2018).

Airways epithelial cells play key role in regulation of lung homeostasis, and maintaining healthy populations of these cells are critical for avoiding lung inflammation. Autophagy has been determined to be essential in the maintenance of epithelial cell counts in pulmonary airways (Li et al., 2019). Simutanously, Inflammatory cytokines, such as TNF-α, IL-1, IL-17, IL-4, IL-13,TGF-β, and so on produced by pathological process. Autophagy and its interactions with these cytokine production largely been reviewed may condition the pulmonary airways and contribute to airway inflammation and airway mucus secretion (Painter et al., 2020). Studies revealed that autophagy is crucial for IL-1β transcription and processing of pro-IL-1β. Autophagy is capable of inhibiting IL-1β production (Harris et al., 2011). Defective autophagy accompanied IL-1 reduction has been shown to reduce IL-1β, decrease BAL neutrophils, and ameliorate lung pathology (Suzuki et al., 2016). And to blocking IL-1R greatly reduced IL-17A and IL-4 (de Luca et al., 2014). IL-17A production contributes to the pathogenesis of asthma. IL-1β and IL-23 were found to induce CD4^+^ lymphocytes’ differentiation into Th17 cells (Wilson et al., 2007). It was also found that IL-1 and IL-23 could induce the expression of IL-17A. IL-17 expression has been demonstrated to augment the expression of Glucorticoid β (GR-β) in epithelial cells *in vitro* (Vazquez-Tello et al., 2010). The function of GR-β is to suppress GR-α-mediated anti-inflammatory gene transcription through a competitive inhibitory mechanism with GR-α (Trevor and Deshane, 2014). The autophagy-related gene5 (Atg5) has been associated with childhood asthma (Martin et al., 2012; Poon et al., 2012). Atg5^−/−^ mice causes severe inflammation and AHR, which is mediated by increased neutrophilic airway inflammation though secretion of IL-17A from T cells (Suzuki et al., 2016). Interleukin (IL)-10 is a key anti-inflammatory cytokine that may be reduced in asthma (Maneechotesuwan et al., 2021). In asthmatic patients, inhibition autophagy enhances IL-10 production, resulting in the control of asthmatic inflammation.

### Autophagy in Mucus Hypersecretion

Ciliated epithelial cells possess up to 300 cilia per cell and a large number of mitochondria are found immediately beneath the apical surface, which are responsible for providing energy to the cilia for mucous clearance up and out of airways via coordinated ciliary beating (Tam et al., 2011). Cigarette smoke (CS) impairs mucociliary clearance (MCC) and epithelial cell cilia shortening (Lam et al., 2013). The function of mucociliary clearance is to maintain an uninfected and unobstructed airway. The impairment of mucociliary clearance might render mucus clearance defective and lead to obstruction. A previous study demonstrated that genetic deletion of the autophagy mediators BECN1 and LC3B, or genetic and chemical inhibition of HDAC6, protects mice from MCC disruption when exposed to CS. The activation of proposed ciliophagy pathway leads to cilia shortening. The excessive activation of autophagy might ultimately lead to ciliated cell loss and ciliated cell death (Cloonan et al., 2014). In addition, the environmental stimulus have shown to induce autophagy activation and subsequently the mucus hypersecretion, including CS and fine particulate matter (Chen et al., 2016; Zhou et al., 2016).

IL-13 plays a key role in modulating the pathology of the onset of asthma (Deo et al., 2010). IL-13 binds to a dimer composed of IL-4 receptor α (IL-4Rα) and IL-13 receptor α1 (IL-13Rα1) so as to regulate the differentiation of epithelial cells to goblet cells (Munitz et al., 2008). Moreover, autophagy activity was associated with mucin-secreting airway epithelial goblet cells that form in response to chronic IL-13 treatment (Dickinson et al., 2016). Atg5 deficiency in human tracheobronchial epithelial cells was found to enhance the accumulation of MUC5AC, suggesting a corresponding functional defect in MUC5AC secretion, but not induce the production of MUC5AC. Another, Atg14 depletion results in reduced MUC5AC secretion following stimulation with IL-13. In addition, Atg5 and Atg14 deficiency reduce IL-13-mediated ROS activity and attenuate IL-13-mediated MUC5AC secretion (Dickinson et al., 2016). These results implied that autophagy pathway is required for both IL-13-mediated MUC5AC secretion and reactive oxygen species (ROS) activity in the airway bronchial epithelial cells (Dickinson et al., 2016). And the underlying mechanism of IL-13-mediated increase in superoxide levels is that autophagy pathway could direct Dual oxidase 1 (DUOX1) to the apical surface of the airway epithelium (Dickinson et al., 2018). Moreover, TGF-β3 activation increased ROS levels in a NOX4-dependent pathway and subsequently induced autophagy as well as MUC5AC expression in the epithelial cells (Zhang et al., 2019). These findings suggest a new mechanism in which autophagy is stimulated to regulate MUC5AC secretion in asthma.

Taken together, autophagy plays a key role in cellular function of a variety of different cell types in different stages of development of asthma focus on different autophagy proteins. Impaired autophagy pathway maybe induced progress of severe asthma. Moreover autophagy can have a direct protective role on epithelial cell populations. However, autophagy contribution in the pathogenesis of allergic asthma through different cells is implicated in the review. Therefore make targeting of autophagy for clinical applications facing challenging.

## The Effects of Chinese Herbal Medicine on Autophagy

**FIGURE 1:**
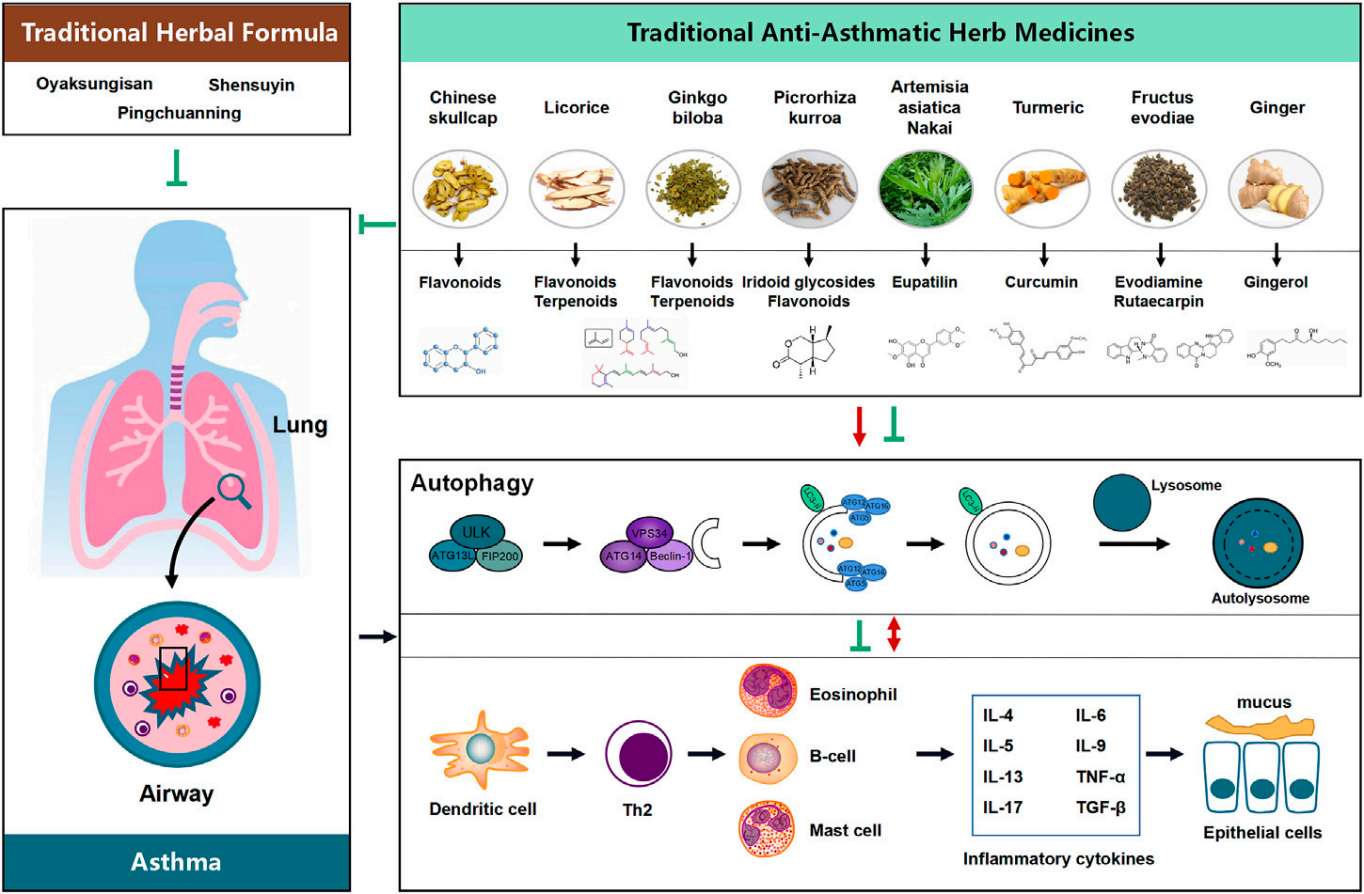
Proposed flow chat involving the influence of the potential benefit of Chinese Herbal Medicine (CHM) in treating asthma possibly via modulating the process of autophagy. Natural products and their active compounds have the potential to modulate autophagy and futher interaction with inflammatory cytokines in the development of asthma, thereby inhibition any possible effects on asthma and ameliorating the development of the diseases.

Therapies targets to autophagy pathway benefit to restore airway inflammation and airway remodeling urging us to find autophagy modulators for treatment of asthma. (CHM), a natural compounds, taking as efficiency source of autophagy modulators come into our sight. Since autophagy pathway underlines a broad range of pathological conditions in asthma, the successful therapeutic outcomes of using autophagy modulators suggest the need for intensively investigating the pharmaceutical potential of compounds with autophagy-adjusting ability. Recent research findings not only shed light on the potential novel applications and formulation of CHMs via regulation of autophagy possibly being an important mechanism underlying the therapeutic effect of CHMs in treating disease, but also highlight that the natural autophagic compounds and extracts from CHM are of interest due to their potential new therapeutic application in diseases. While a number of autophagy regulators, such as Oyaksungisan, Shensuyin, Ginseng, Turmeric and Ginger have been reported, natural autophagic compounds from CHMs are of interested because of their potential new therapeutic applications (Figure 1).

### The Herbal Medicine Formula in Modulating Autophagy

CHM is an ancient yet still active medicinal system widely used in China. A major therapeutic approach of CHM is the use of a mixture of herbs, called the Traditional Medicine Formulation. The mixture of herbs exerts therapeutic actions and modulates other effects, with the principal herbal constituents providing the main therapeutic actions.

Oyaksungisan (OY) is a traditional herbal medication formula consisting of twelve herbs: Ephedra Herb, Citrus Unshiu Peel, Lindera Root, Cnidii Rhizoma, Angelica Dahurica Root, Batryticatus Bombyx, Aurantii Fructus Immaturus, Platycodon Root, Zingiberis Rhizoma, *Glycyrrhizae* Radix et Rhizoma, Zingiberis Rhizoma Crudus, and Zizyphi Fructus (Yim et al., 2013). Some of the herbs in OY, including Citus Unshiu Peel, Lindera Root, Angelica Dahurica Root and Zingiberis Rhizoma, have been reported to have anti-cancer effects through induction of autophagy (Yim et al., 2013). OY can induce the activation of Mitogen-activated protein kinase (MAPK) cascades involved in the activation of autophagy also via JNK activation. JNK signaling pathway accompanying with autophagy activation might contribute to the maintenance of allergic airway inflammation (Fang et al., 2020). Numerous studies have reported the bioactivities of OY of neuroprotection, anti-H_2_O_2_-induced apoptosis and anti-inflammation effects (Oh et al., 2012; Yim et al., 2013). OY contains anti-inflammatory activity related with inhibition of NF-κB pathway (Oh et al., 2012). NF-κB signaling pathway and autophagy pathway have the potential to modulate asthma (Song et al., 2019). These indicated that OY have effect of modulating autophagy potentially influence in inflammation of asthma.

Pingchuanning decoction is a well-known traditional Chinese medicine for the treatment of airway inflammatory diseases. Pingchuanning decoction had an intervention effect on alleviation of airway inflammation, relief of bronchial smooth muscle spasm, and inhibition of airway remodeling in asthma (Fang et al., 2012). Pingchuanning decoction contributes to the amelioration of airway inflammation via inhibition of autophagy (Wang et al., 2019).

Shensuyin (Samsoeum, SSE), a traditional herbal formula, has been widely used to treat cough and fever (Kim et al., 2013). The herbal plants in SSE include Paerillae Folium, Puerariae Radix, Pinelliae Tuber, Angelicae Decursivae Radix, Ginseng Radix, Poria Sclerotium, Autantii Fructus Immaturus, Platycodonis Radix, *Glycyrrhizae* Radix et Rhizoma, Citri Unshius Pericarpium, Zingiberis Rhizoma Crudus, and Ziziyphi Fructus. Recent studies have reported that SSE can modulate asthma reactions and pulmonary damage via inhibition of the expression of cyclooxygenase 2 (COX-2), of inflammatory cytokines and of the activation of NF-κB (Cho et al., 2008; Kim et al., 2013). SSE has a potential function targeting to autophagy pathway in modulating cell death and G2/M arrest in cancer cells. SSE treatment can significantly increase the ratio of LC3II to LC3I in cancer cells. Moreover, SSE treatment can inhibit the PI3K/Akt/mTOR signaling pathway. PI3K/Akt/mTOR pathway inhibition is consistent with the induction of autophagy. Moreover, SSEW inhibited the infiltration of inflammatory cells, hyperplasia of goblet cells, and the expression of iNOS in allergic asthma asthma (Jeon et al., 2015a). Thus SSEW has anti-asthma and targeting to autophagy properties.

### The Single Herbs in Modulating Autophagy

For the past several thousands of years, Traditional Herbal Formulation has been used to prevent and treat a number of disorders. But to find the directed association between scientific proof and molecular analysis of CHM, a growing number of studies of CHM in disease have focused on molecules extracted and purified from individual herbs. At present, a number of individual herbs have been reported to be beneficial to curing disease via modulating autophagy, such as Radix Ginseng (Ren Shen), Ridix Sophorae Flavescentis (Ku Shen) and others.

Ginseng has been prescribed for maintaining the bioenergetics balance, as suggested by Chinese herbalists, for breathlessness and diabetes. Rb1, Rg1, Rg3, Rh1, Re, and Rd are bioactive ginsenosides from Ren Shen that were revealed by pharmacological studies (Chen et al., 2008). The traditional use of ginseng focuses on its autophagic mechanisms in suppressing neurotoxicity, enhancing cardiac muscle cell survival and suppressing breast cancer stem cells as well as hepatocellular carcinoma. Recent research has reported that gensenosides could also ameliorate inflammation and stimulate the immune system. This research implies a potential relationship between the role of autophagy and the role of modulating inflammation by ginseng, suggesting autophagy in the potential new therapeutic action of ginseng in modulating the onset of asthma.

Ridix Sophorae Flavescentis (Ku Shen), documented in editions of the pharmacopoeia of the People’s Republic of China, has had more than 200 compounds isolated and extracted. Modern pharmacological studies have shown that matrine is the most active of these components with multiple pharmacological effects, including anti-tumor, antiviral, and anti-inflammation, as well as anti-asthmatic activities (Wang et al., 2015a; You et al., 2020). The main target of matrine’s anti-tumor therapeutic effects is to induce autophagic cell death and apoptosis in the cancer cells. Matrine treatment can induce apoptosis in gastric cancer MKN45 cells, leukemia U937, and K562 cells, and C6 glioma cells (Liu et al., 2006; Jiang et al., 2007; Luo et al., 2007; Zhang et al., 2009). Both autophagy and apoptosis are activated in HepG2 cells and gastric cancer SGC-7901 cells following matrine treatment (Zhang et al., 2010; Li et al., 2013). Matrine treatment in HepG2 cells and gastric cancer SGC-7901 cells up-regulates mRNA expression of Beclin1, which is a critical component of mammalian autophagy and a haploinsufficient tumor suppressor gene. The present pharmacological studies have revealed that the anti-inflammation of matrine is related to the inhibition of ROS production and oxidative stress, and the inhibition of NF-κB activation.

At present, there are occurring a lot of discoveries of natural autophagy regulators from CHM with potential use in therapeutic applications in cancer and neurodegenerative disease models. Although little direct linkage between the traditional herbs-induced autophagy and its anti-asthmatic effects has been reported, owing to the significant role of autophagy in immunomodulation, the involvement of such a process cannot be disregarded.

### Traditional Anti-asthmatic Herbal Medicines

#### Licorice, the Root of *Glycyrrhiza* Uralensis

Licorice, the root of *Glycyrrhiza* uralensis, is one of the most frequently used drugs in traditional Chinese medicine. A large number of prescriptions contain licorice as a significant component present in Shang-Han-Lun (Katakai and Tani, 2003). Licorice possesses properties with potential benefit for asthma suffers. Some traditional formulas containing licorice, for example, “Shaoyao-Gancao-tang”, have been prescribed for treating asthma (Shibata, 2000). Pharmacological studies have revealed that the major bioactive components in licorice root are flavonoids and pentacyclic triterpene saponin (Kamei et al., 2003). Isoliguiritigenin is a member of the flavonoids, which evokes obvious tracheal relaxation effects Liu et al. (2008), and the traditional use of licorice for asthma treating is due to anti-inflammation (Kuang et al., 2018; Huang et al., 2019; Upadhyay et al., 2020) related to inhibiting inflammatory cells’ infiltration, decreasing oxidative stress, and reducing pro-inflammatory mediators’ production, such as TNF-α and IL-1β (Yu et al., 2018; Hou et al., 2019). In addition, licorice and its component licochalcone-A can induce autophagic cell death though inhibition of the mTOR pathway in cancer cells (Yo et al., 2009). These findings shed light on the development of the new usage of licorice in disease treatment through induction of autophagy. However, the mechanism of autophagy in mediating the traditional anti-asthmatic function of licorice remains to be investigated.

#### Chinese Skullcap (Huang Qin)

Chinese skullcap (Huang Qin) is a common herbal remedy in the field of traditional medicine. The root of skullcaps, known as Radix Scutellariae, is the source of the Chinese medicine Huang Qin (Zhao et al., 2019). It has been in use for over 2000 years as a remedy for the treatment of hepatitis, diarrhea and inflammatory diseases such as asthma (Zhao et al., 2016). Pharmacological studies have revealed that the biological activities in Huang Qin originate from flavonoids like baicalein, baicalin, wogonin, and wogonoside (Li-Weber, 2009). The biological compounds of Huang Qin can help prevent the histamine discharge from mast cells *in vitro*. Baicalin was shown to effect anti-asthmatic activity in isolated tracheal muscle from asthmatic guinea pigs (Liaw et al., 1999). Current research on the effect of Chinese Skullcap in asthma is limited to alleviating the inflammation and hypersensitivity prevailing in the airways. There is no precise mechanism known of the anti-inflammation function of Chinese Skullcap in asthma. The anti-cancer effect of Radix Scutellariae, such as induction of cell death and cell cycle arrest, could be mediated through autophagy (Wang et al., 2015b). Although the involvement of autophagy in the Radix Scutellariae-triggered anti-asthmatic disease mechanism is still elusive, the progression of gastroenteritis and hepatitis are highly autophagic-related, suggesting a potential autophagic therapeutic role by Radix Scutellariae in chronic inflammation diseases such as asthma.

#### Turmeric (Jiang Huang Su)

Turmeric (Jiang Huang Su) possesses a wide spectrum of biological and pharmacological activities (Sharifi-Rad et al., 2020). Curcumin is a component of turmeric, which has been used for centuries in traditional Chinese medicine to treat several diseases (Jennings and Parks, 2020; Li et al., 2020). The biological effects of curcumin, such as anti-inflammation and anti-oxidant effects, have been reported in asthma treatment (Lelli et al., 2017). The anti-inflammatory effect of curcumin in treating asthma not only mediated the expression of inflammatory factors but also regulated many inflammatory cells involved in modulating NF-κB pathway and downregulation of inflammatory cytokines such as IL-5 and IL-8 (Lelli et al., 2017). Treatment of an asthmatic model of mice with curcumin leads to significant reduction of eosinophils, neutrophils monocytes and mast cells so as to blocking histamine release (Ammar et al., 2011; Abidi et al., 2014), which meanwhile inhibiting airway smooth muscle cell proliferation and ameliorating airway remodeling in asthma (Kobayashi et al., 1997; Zeng et al., 2013; Chauhan et al., 2014). Recently, curcumin has been found to have therapeutic potential towards cancer by autophagy pathway. (Kim et al., 2012; Xiao et al., 2013). Also, curcumin has autophagic modulating properties via mediating the PI3K/Akt/mTOR pathway in neuroprotective and renoprotective effect (Wang et al., 2014). These findings suggest that autophagy may be behind the newly discovered properties of curcumin targeting chronic inflammatory diseases, including asthma.

#### Artemisia Asiatica Nakai (Ai Cao)

Artemisia asiatica Nakai (Ai Cao), which belongs to the family Compositae, specifically the leaves of this plant, is known for its anti-inflammatory, anti-microbial, anti-tumor, anti-oxidative, neuroprotective, and gastroprotective properties, has been documented to be effective in various disease conditions (Lee et al., 2008; Lim et al., 2008; Jeong et al., 2014; Ahuja et al., 2018). Eupatilin is regarded as the main phytochemical and bioactive component of the flavonoids from this plant (Jung et al., 2012). Eupatilin plays a therapeutic role in asthma, inhibiting the infiltration of inflammatory cells such as the inhibition of adhesion of eosinophils to bronchial epithelial cells (Jeon et al., 2015b; Ahuja et al., 2018). DA-9601, a Food and Drug Administration-approved drug that comprises eupatilin as an active ingredient, was developed as a clinical therapeutic agent for asthma treatment due to its capability of suppressing the airway allergic inflammation via regulation of various cellular molecules expressed by the MAP kinases/NF-κB pathway (Kim et al., 2006). DA-9601 can also reduce tissue remodeling by inhibiting TNF-α-induced MMPs and goblet cell hyperplasia caused by IL-5 (Kim et al., 2006). Eupatilin also induces autophagy against arachidonic acid and iron-induced oxidative stress in HepG2 cells (Jegal et al., 2016). However, little research has reported the role of autophagy induced by eupatilin in mediating allergic airway inflammation. These findings suggest that autophagy may be the underlying molecular mechanism responsible for treating asthma by artemisia asiatica.

#### Picrorhiza Kurroa (Hu Huang Lian)

Picrorhiza kurroa (Hu Huang Lian) is a small perennial herb belonging to the family scrophulariaceae, and is a well-known herb of traditional medicine with anti-oxidant, anti-inflammatory, and immunomodulatory activities (Russo et al., 2001). The rhizome of P. kurroa has been reported to contain iridoid glycosides, such as picroside I and picroside II, terpene like cucurbitacins and flavonoids like apocynin, which are responsible for the anti-cancer, anti-asthmatic and hepatoprotective potential of the plant (Zahiruddin et al., 2017). The anti-asthmatic effect of Picrorhiza kurroa reduces airway obstruction in asthma (Anand et al., 2008). Previous studies have reported that the anti-inflammatory activity of Picrorhiza kurroa is mediated through the suppression of macrophage-derived cytokines and mediators via suppression of NF-κB signaling (Piao et al., 2017). At present, there is little knowledge of the precise mechanism of Picrorhiza kurroa in mediating allergic airway inflammation (Mahajani and Kulkarni, 1977). Picroside II is one of the most effective components extracted from P. kurroa, which affects the autophagic pathway in modulating the progression of severe acute pancreatitis (SAP) via inhibiting NF-κB, TNF-α, and SIRT1, suggesting that the autophagy pathway provides a new insight into asthma treatment using Picrorhiza kurroa.

#### Ginkgo Biloba (Yin Xing)

Ginkgo biloba (Yin Xing) has been used in traditional Chinese medicine for about 1,000 years. Ginkgo biloba extract (GBE) is collected from the dried green leaves of the plant (Singh et al., 2019). Pharmacological studies have demonstrated that Ginkgo biloba extract contains two major active components including flavonoids (ginkgo-flavone glycosides) and terpenoids (ginkgolides and bilobalides) (Loew, 2002). The therapeutics effect of GBE in asthma has been reported to present as a decrease of vasopermeability, bronchoconstriction relief and the amelioration of patients’ hypersensitivity to antigens (Tang et al., 2007). However, there is a lack of definitive evidence and direct mechanism that GBE works in improving the asthma condition. The traditional use of GBE is as a complementary and alternative medicine option for neurodegenerative diseases via the autophagy pathway (Liu et al., 2015b), suggesting that autophagy might be a new choice mechanism of GBE treatment for other diseases, such as asthma.

#### Fructus Evodiae (Wu Zhu Yu)

Fructus evodiae (Wu Zhu Yu) has been used frequently as a traditional medicine against inflammatory diseases in China and Japan. Evodiamine (EVO) and rucarpine are compounds extracted from Fructus evodiae, which were shown to have inhibitory effects on TNF-α and IL-4 protein expression in RBL-2H3 cells induced by IgE-antigen complex (Yang et al., 2018). These results imply that EVO and rutaecarpine may be effective against IgE-induced allergic diseases. EVO is a major alkaloid compound extracted from the dry unripened fruit Evodiae fructus (Evodia rutaecarpa Benth., Rutaceae). EVO has a variety of pharmacological activities, such as anti-obesity, anti-allergenic, analgesic, anti-tumor, anti-ulcerogenic, and neuroprotective activities (Tan and Zhang, 2016).

#### Ginger (Sheng Jiang)

Ginger (Sheng Jiang) has been used in traditional medicine for the treatment of respiratory diseases such as asthma (Yocum et al., 2020). Pharmalogical activities of ginger and its constituents in health management work through modulation of various biological activities, including antioxidant activity, anti-inflammatory activity, anti-tumor activity, anti-microbial activity, anti-diabetic activity, and neuroprotective, gastroprotective, hepatoprotective effects (Mao et al., 2019). The active compounds of ginger comprise of gingerol and other gingerol-related compounds, paradols, shogaols, zingerone, zerumbone, 1-Dehydro- (10) gingerdione, terpenoids, and genger flavonoids (Chrubasik et al., 2005). A previous study has shown that ginger and its isolated active components may provide a therapeutic option, alone or in combination with accepted therapeutics such as β2-agonists, in asthma through modulation of the relaxation of airway smooth muscle (ASM) and attenuating airway hyperresponsiveness, in part by altering intracellular calcium regulation (Townsend et al., 2013). Ginger extracts can arrest allergic inflammation in the airway though inhibiting inflammatory cell infiltration in blood and BALF, decreasing IL-4 and IL-5 expression in the lung tissues and BALF (Khan et al., 2015). In addition, 6-gingerol was found to induce cervical cancer cell death by upregulating caspase-3-mediated apoptosis and autophagy, partly via the repression of Akt signaling (Chakraborty et al., 2012). In pancreatic cancer, 6-gingerol induces cytotoxicity exclusively through autophagy by activating AMPK-mTOR signaling, which is a process independent of necroptosis and apoptosis (Law et al., 2016). These findings suggest potential autophagy properties of Ginger, which supports the possibility of a novel mechanism of autophagy modulating-activity of Ginger exhibiting therapeutic effects towards asthma.

Bronchial inflammation and airway remolding are common pathological features shared by asthma. However airway remolding the major difficult in treatment accompaning decreasing of lung function characteristiced smooth muscles rewalling, globet cell hyperplasia and mucus hypersecretion, which is induced extensively, though not exclusively. These asthma therapeutic compounds listed here were reported mostly by their anti-inflammation features along with relaxation of airway smooth muscle (ASM), attenuating airway hyperresponsiveness and inhibiting inflammatory cell infiltration. Another facet to consider for asthma therapy with these proposed potential traditional medicines is their polypharmacological nature. For example, they are also autophagy modulators in cancer or neurodisease, reinforcing that these CHMs potentialy simultaneously target autophagy passway focused on anti-inflammation and futhur anti-mucus hypersecretion via interaction with inflammation cytokines.

## Conclusion

Above all, CHM is one of the main lines of complementary and alternative therapy of bronchial asthma, as it is the third most popular choice of both adults and children suffering from this condition. An increasing number of CHMs have been discovered as autophagy modulators. Such autophagic-regulatory effects are potentially therapeutic for allergic inflammatory diseases such as asthma. Although we have found that many autophagy modulators isolated from anti-asthmatic-CHMs were possibly related to new mechanisms and functions of these plants in the treatment of asthma diseases, we are still far away from translating these traditional herbs or formula into clinical applications in asthma therapy. The cell type-specific property of autophagy should be taken into account, which may otherwise minimize the efficacy of the applied herbs. Moreover, the functional consequences of autophagy induction on these cells vary under different disease conditions. Therefore, detailed and systematic investigations concerning the interaction between CHMs and autophagy-related disorders in a comprehensive molecular approach are needed. Positive findings in these areas could widen the scope of CHM applications by suggesting novel intervention strategies, which have not been mentioned in the traditional Chinese pharmacopeia.

Since the molecular mechanisms and functions of many autophagy modulators isolated from CHM have been intensively studied, we will work in the future to provide a direct link from traditional use to new pharmacological applications associated with autophagy in asthma treatment with these plants.

## Prospect

Taken to note, as the certein mechanisms of either of the CHMs list in the paper modulates autophagy in response to asthma related stimulis may vary depending on the type of cells or the cell metabolism and/or environment Thus we need to further studing asthma therapeutic drugs or autophagy modulators conducted on asthma models *in vivo* and invtro to reveal the polypharmacological approach of natural products taget the diseases with complex pathologies such as asthma.
